# Development of D-α-Tocopherol polyethylene glycol 1000 succinate fabricated nanostructural lipid carrier of sorafenib tosylate for metastatic colorectal targeting application: Stability, physical characterization, cytotoxicity, and apoptotic studies against SW48 cells PTEN

**DOI:** 10.3389/fonc.2022.990841

**Published:** 2022-10-26

**Authors:** Sankha Bhattacharya, Satyam Sharma, Bhupendra G. Prajapati

**Affiliations:** ^1^ Department of Pharmaceutics, School of Pharmacy & Technology Management, SVKM’S NMIMS Deemed-to-be University, Shirpur, Maharashtra, India; ^2^ Department of Pharmacology and Toxicology, National Institute of Pharmaceutical Education and Research (NIPER), Export Promotion Industrial Park (EPIP), Hajipur, Bihar, India; ^3^ Department of Pharmaceutical Technology, Shree S.K. Patel College of Pharmaceutical Education & Research Ganpat University, Gujarat, India

**Keywords:** sorafenib tosylate (ST), SW48 Cells PTEN, apoptosis assay, colorectal cancer, D-α-Tocopherol polyethylene glycol 1000 succinate (TPGS), Nanostructural Lipid Carrier (NLC)

## Abstract

The study aimed to create D-α-Tocopherol polyethylene glycol 1000 succinate (TPGS) nanostructured lipid carriers (NLC) of sorafenib tosylate (ST) as lymphatic delivery systems (LDDS) to fight Metastatic colorectal cancer. Initially, ST-SLN, ST-NLC, and ST-LNE were formulated considering oleic acid (OA), glycerol monolinoleate (GMO), glycerol monolinoleate (GML) as solid lipid and further characterised, and tested for stability. The most stable ST-NLC was fabricated with TPGS to produce ST-TPGS-NLC and evaluated by performing *in vitro* drug profiling, *in vitro* cytotoxicity, and apoptotic studies against human female colorectal adenocarcinoma cell lines (SW48 Cells PTEN). Stability studies on three lipidic nanoparticles (ST-SLN, ST-NLC, ST-LEN) showed particle size, polydispersity index, and zeta potential ranging from 165 nm to 298 nm, 0.125 to 0.288, and -31 mV to -16 mV. At 1600 minutes, more than 80% of ST-NLC1 was released, confirming the sustained release pattern of the formulation. ST-NLC and ST-TPGS-NLC have entrapment efficiencies above 50%. Pure ST’s IC50 at 72 hr was 3.45 µg/mL, while 1.56 µg/mL was for ST-TPGS-NLC. The ST-TPGS-NLC reduced the number of livings SW48 Cells PTEN from 91% to 5%, compared to 75% to 8% of pure ST. The ST-TPGS-NLC is a promising LDDS for delivering ST for metastatic colorectal cancer.

## 1 Introduction

Due to the recent advanced research on nanoscale particular system (20-1000nm) it was finally reviled that nanoparticles are actually transported through lymphatic system rather than blood capillaries (1). To achieve enhanced lymphatic drug delivery system (LDDS), more advanced fundamental research is going on in the filed of lipid-based nanoparticles, dendrimers, liposomes, polymeric nanoparticles (2). In more conventional way to deliver medicines through LDDS approach, dermal injection is a promising approach (3, 4). However, nanoscale materials are capable of crossing stratum corneum; which is basically a barrier which stops topically applied medicine from getting to where it needs to go. Also, medicines that are put on the skin don’t have to go through the liver’s first-pass metabolism, which makes them more effective. Additionally, medications administered topically bypass the liver’s first-pass metabolism, increasing their potency. Recent studies explain the significant importance of nanoscale lipid-based drug carriers as lymphatic drug delivery systems (LDDS) as they can easily move through the lymphatic system. Shigeki Kato et al. (5) used ultrasound to improve the way drugs are delivered through the lymphatic system. They found that molecules were more likely to get to the target lymph node if they were used for an upstream lymph node (LN). From this study, it was clear that lymphatic drug delivery systems (LDDS) could be used to treat many kinds of diseases (5).Cancerous cells frequently migrate through lymph nodes, LDDS can be extremely effective in preventing the spread of cancer to other body organs (6). Furthermore, LDDS could guarantee that all cancerous cells, including those that escaped through lymph nodes and may develop into cancer, are eradicated from the body. Chemotherapeutic drugs can be transported by nanoscaled lipid-based materials like liquid nano emulsions (LEN) (7), solid lipid nanoparticles (SLN) (8, 9), and nanostructural lipid carriers (NLC) (10). Nerolidol-loaded solid lipid nanoparticles (NR-LNPs), created by Shabi Parvez et al. (11) were found to increase the bioactive’s solubility and stability. The study also shown that Caco-2 cells may quickly internalise NR-LNPs. Additionally, Caco-2 cells’ apoptosis can be greatly increased by NR-LNPs (11). These lipid-based formulations with nanoscale sizes work as nanocarriers that travel through the lymphatic system the same way lipoproteins do to be absorbed. The main difference between SLN, LEN, and NLC is the lipid core (12).

The lipid core of LEN is liquid oil, the lipid core of SLN is solid oil, and the lipid core of NLC is both solid and liquid oil (13). These nanoscale systems on a large scale are safe to use around living things because they are made of lipids and break down over time (9). Poor lymphatic drainage and the Enhanced permeability and retention effect (EPR) also make it more likely for nanoscale drug payload to build up in tumour tissue (14). According to Andrew D Wong et al. (15), aberrant tumour vasculature can cause inadequate lymphatic drainage. Enhanced permeation and retention (EPR) refer to the increased permeability that mediates medication uptake in solid tumours. By using a pharmacokinetics model, the scientists showed how the EPR effect affects tumour uptake using doxorubicin from human treatment trials (15). It’s interesting that the engineering of lipid-based formulations at nanoscales has a big effect on how well therapies work (16). This happened because the drug got to the place where it worked better and systemic toxicity went down. For example, coating nanoparticles with polyethylene glycol (PEG) stops macrophages from opsonizing and eating them. Because of this, there is a better chance that the drug will get to the lymphatic system and the mass of the tumour and that it will stay in the body longer (17). Also, the receptors caveolin and clathrin are more visible on the surface of cancerous cells. Because of this, caveolin and clathrin-mediated endocytosis can be used with negatively engineered nanoparticles to deliver cancer (18). Additionally, it was known that cancer cells can more easily absorb vitamin E-coated nanoparticles due to the overexpression of their receptors (19). The biodistribution study also revealed that Colorectal cancer concealed a significant amount of drug-loaded lipid-based nanoparticles made of stearic acid (20). Making nanoscale lipid-based formulations enhances the LDDS of administered chemotherapeutic agents. To confirm this topic, more research must be conducted. The term colorectal cancer (CRC) refers to the development of cancer in the colon, rectum, or large intestine (21). This condition’s occurrence can be linked to an abnormally high rate of cell division, which has the potential to spread or invade other parts of the body. The third most common type of cancer in both men and women is colorectal cancer (CRC) (22). The highest incidence of colorectal cancer is seen in people between the ages of 60 and 79. Only about 20% of incidents involve victims who are under the age of fifty. Every region of the world has a risk of developing colorectal cancer, but the countries of Eastern Europe, Australia, and the United States have the highest mortality rates. Sorafenib tosylate (ST) belongs to the group of drugs known as kinase inhibitors. It functions by preventing the action of a troublesome protein that instructs cancer cells to proliferate. Sorafenib tosylate (ST) can be used to treat CRC. Sorafenib tosylate has been shown to interact with a wide range of kinases, including those on the cell surface as well as those found inside the cell (KIT, FLT-3, VEGFR-2, VEGFR-3, and PDGFR-ß), CRAF and BRAF, as well as mutant BRAF. It’s possible that some of these kinases play a role in the angiogenesis process (23). Sorafenib works in this manner to lessen blood flow to the tumour (24). Sorafenib tosylate (ST) is a special drug because it blocks the Raf/Mek/Erk pathway (25). Genetic transcription that is involved in both cell proliferation and angiogenesis can be stopped by inhibiting these kinases. In order to combat the lymphatic metastasis of colorectal cancer, the current study sought to engineer D-α-Tocopherol polyethylene glycol 1000 succinate (TPGS) nanoscale Sorafenib tosylate (ST) loaded lipid-based as LDDS of ST (26). To increase the anticancer effect in colon cancer cells, Yanlei Wang et al. (27) created oxaliplatin (OXL)-loaded D-α-Tocopherol polyethylene glycol 1000 succinate (TPGS) based lipid nanoparticles. The anticancer impact of OXL was noticeably enhanced in HT-29 colon cancer cells by the presence of TPGS. Free OLX had an IC50 of 4.25 µg/ml, but OXL-loaded TPGS-based lipid nanoparticles (OXL/TLNP) had an IC50 of 1.12 µg/ml. The enhanced anticancer impact of OXL based on nanoparticles is demonstrated by the 3-fold lower IC50 value of OLX/TLNP. The findings signify the importance of TPGS in cancer research (27). When creating drug delivery systems, D-alpha-tocopheryl polyethylene glycol succinate (TPGS) is widely utilised to enhance the pharmacokinetics of anti-cancer medications and lower multi-drug resistance (28). Pharmaceutical characterization was performed on ST liquid nano emulsion (ST-LNE), ST nanostructured lipid carriers (ST-NLC), and ST solid lipid nanoparticles (ST-SLN). To find the ideal formulation, *in vitro* dissolution was applied to the best lipid-based nanoparticles. Additionally, stability was investigated in order to choose the more stable lipid-based nanoscale carrier. In addition, the TPGS-engineered optimised formulation of NLC (ST-TPGS-NLC) was tested for cytotoxicity and apoptosis against human female colorectal adenocarcinoma cell lines (SW48 Cells PTEN) to serve as a surrogate model for colorectal cancer.

## 2 Materials and methods

### 2.1 Materials

Neon Pharmaceutical Ltd., Mumbai, India, provided a gift sample of sorafenib tosylate (ST). Thermo Fisher Scientific, India, provided PluronicTM F-68 Non-ionic Surfactant (100X) (PF-68, HLB: 29) and D-Tocopherol polyethylene glycol 1000 succinate (TPGS). Sigma Aldrich – Merck, Bengaluru, India, provided stearic acid (SA, molecular weight 284.48 g/mol) and oleic acid (OA long-chain fatty acid; LCFA), 30 mg/g (balance methyl—cyclodextrin). Sigma Aldrich-Merck, Bengaluru, India, provided long-chain Glycerol monolinoleate (GMO) and Maisine 35-1 (glycerol monolinoleate; GML, long-chain monoglyceride; LCM). Thermo Fisher Scientific in India provided the L-phosphatidylcholine.

### 2.2 Solubility study

Sorafenib tosylate (ST) solubility in different types of solid lipids was studied visually as previously described by Ladan Dayan et al.,(2022) (29). To check solubility, 200 mg of each solid lipids and 50 mg of ST mixed in vial and heated up to 75°CC. An aliquot amount of drug-ST added further if the initial drug gets dissolved. As per Rosa MariaIacobazzi et al., If 50mg drug failed to dissolve than once again further amount of solid lipids were added till the formation of clear solution (30). Briefly, an excess amount of ST was placed in a screw-capped glass vial containing approximately 1 gm lipids (SA, OA, or GMO) and magnetically stirred for 48 hours at room temperature to determine ST solubility. The mixture was centrifuged for 15 minutes at 12500 rpm at the end of the experiment, and the drug concentration in the supernatant was determined using the developed RP-HPLC method. ST solubility in each oil was determined three times.

### 2.3 Preparation of SLN, NLC, and LE

Surajit Das et al. (31) used the ultrasonic melt-emulsification method to prepare lipid-based formulations, with minor changes. **Table 1** explain how to make each formulation. In distilled water, a predetermined amount of surfactant and tocopheryl polyethylene glycol succinate (TPGS) (in the case of P-SLN) were mixed together to make an aqueous phase. In a cylindrical beaker, weighted amounts of solid lipid were added without (Plain-SLN or Plain-P-SLN) or with ST (ST-SLN or ST-P-SLN). Both beakers are preheated to 80°C at the same time during the preparation. The liquefied lipid beaker was placed over an 80°C preheated Magnetic-Stirrer heater, and the hot aqueous phase was gradually added. To make the primary microemulsion, add the magnetic stir and increase the mixing speed to 5000 rpm for 3-5 minutes. Following that, SLN was obtained from a primary hot microemulsion using an Ultrasonic Cell Disruptor, USCG-300 (Bioevopeak Inc.USA) at 85 percent voltage efficiency for 6 minutes, with each cycle lasting 20 seconds and a 5 second resting period. The SLN was immediately placed in the fridge to cool.

**Table 1:** Composition of the prepared Primary-SLN, ST-SLN, Primary-NLC, ST-NLC, Primary -LE, and ST-LE.

Formulation code	Lipid phase (mg)		Aqueous phase (mg)		Drug (ST) (mg)
	SA	OA	PF-68	TPGS	ST
Primary-SLN_1_	1200		400		
Primary-SLN_2_	1200		400		
Primary-SLN_3_	1200		400		
ST-SLN_1_	1200		400		80
ST-SLN_2_	1200		400		80
ST-SLN_3_	1200		400		80
Primary -NLC_1_	1000	500	400		
Primary -NLC_2_	1000	500	400		
Primary -NLC_3_	1000	500	400		
ST-NLC_1_	1200	500	400		80
ST-NLC_2_	1200	500	400		80
ST-NLC_3_	1200	500	400		80
ST-TPGS-NLC	1000	700	400	70	80
Primary -LNE_1_		1000	400		
Primary -LNE_2_		1000	400		
Primary -LNE_3_		1000	400		
ST-LNE_1_		1000	400		80
ST-LNE_2_		1000	400		80
ST-LNE_3_		1000	400		80

The amount was given in milligrams. The predetermined amount of surfactant was dissolved in 50 g of distilled water as a continuous phase in all formulations. Primary-SLN (drug free-solid lipid nanoparticle), ST-SLN (sorafenib tosylate (ST)-loaded solid lipid nanoparticle), Primary-NLC (drug free-nanostructural lipid carrier), ST-NLC (sorafenib tosylate (ST)-nanostructural lipid carrier), ST-P-NLC (sorafenib tosylate (ST)-loaded PEGylated nanostructural lipid carrier), Primary-LNE (drug free-liquid nano emulsion), ST-LNE (sorafenib tosylate (ST)- liquid nano emulsion), SA (stearic acid), OA (oleic acid), PF-68 (Pluronic F-68), TPGS (D-α-Tocopherol polyethylene glycol 1000 succinate).

### 2.4 Physicochemical characterization

#### 2.4.1 Particle size, polydispersity index, and zeta potential

A Delsa Nano C photon correlation spectroscopy (PCS) (Beckman Coulter, USA) was used to measure particle size (PS), polydispersity index (PDI), and zeta potential (ZP) for each formulation. Each formulation was diluted in distilled water (1: 1000) and tested at 25°C. The principles of Dynamic Light Scattering (DLS) and Laser Doppler Velocimetry (LDV) modes utilized to measure (Particle size beside PDI) and ZP, respectively. The discovered values calculated as a three-measurement average, with each value being reported as a six-measurement average.

#### 2.4.2 DSC

ST-SLN_1_, ST-SLN_3_, and ST-NLC_1_, ST-NLC_3_ were subjected to DSC analysis using the SHIMADZU DSC-60 Plus Series Differential Scanning Calorimetry, Japan, in the temperature range of 30-210°C at 20 and 100°C/min. ST-SLN_1_, ST-SLN_3_, and ST-NLC_1_, ST-NLC_3_ were analysed using the DSC8000 Perkins Elmer (Waltham, MA, USA) apparatus in the temperature range of 25-205°C at two different heating rates of 20 and 100°C/min. On this apparatus, an autosampler and chiller were installed, and the samples were evaluated with a nitrogen purge at around 20 mL/min. Each sample weighed 3 mg and was placed in an aluminium pan that was tightly sealed.

#### 2.4.3 PXRD

After preparing SLN, the PXRD spectra of ST, SA, freshly melted and cooled SA, PF-68, ST-SLN_1_, ST-SLN_3_, and ST-NLC_1_, ST-NLC_3_ were taken in order to evaluate the molecular state of SA and ST crystallinity. A benchtop X-ray diffractometer (XRD) instrument (MiniFlex; Rigaku) was used for the X-ray diffraction analysis with a scanning rate of 0.5/min in the scanning range of 3-180°C. This instrument was used to carry out the research that was presented. The characteristic peak of each sample was evaluated by collecting the data using monochromatic radiation (Cu Kα´ 1, λ = 1.54 Å), with the voltage set at 40 kV and the current set at 40 mA. This process was carried out under operating conditions of 40 kV and 40 mA.

### 2.5 Measurement of nanoparticles surface morphology

#### 2.5.1 Scanning electron microscopy and transmission electron microscopy

Scanning electron microscopy (SEM) on the prepared nanoparticles was carried out with a JSM-IT800 Field Emission Scanning Electron Microscope (Tokyo, Japan). Fixed and sputtered samples are observed at a voltage of 20 kV on gold-palladium metal plates with a thickness of 100Å. The morphology of Primary-SLN3, ST-SLN3, Primary -NLC3, ST-NLC3, ST-TPGS-NLC, Primary-LNE3, ST-LE3 was determined by using TEM electron microscopy (Hitachi 7500, Japan). Before TEM analysis, the nanoparticles were coated in carbon and placed on a copper grid stained with 1% phosphotungstic acid. The developed images from TEM studies are interpreted by software that generates digital micrographs.

#### 2.5.2 Atomic Force Microscope

An Atomic Force Microscope (AFM) can characterise Primary-SLN3, ST-SLN3, Primary-NLC3, ST-NLC3, ST-TPGS-NLC, Primary-LNE3, ST-LE3 nanoparticles in 3D with sub-nanometer resolution. AFM research was carried out in the Hitachi AFM5300E under unusual circumstances. AFM experiments can be carried out at temperatures ranging from -120 to 800°CC. The AFM probe has two dimensions of movement: length and width. One drop of the nanosuspension of various nanoparticles was dropped into the microscopic sender slide and allowed to dry at room temperature. Based on the 3D images that were created, the roughness and kurtosis average parameters were calculated.

#### 2.5.3 Encapsulation efficiency

The indirect method was utilised in order to determine the EE % of the Sorafenib tosylate (ST) in the drug-loaded SLN. To summarise, in order to precipitate loaded SLN, a specific quantity of the prepared formulation was centrifuged for forty minutes at a speed of sixty thousand revolutions per minute (32). Using the newly developed UV-UPLC method, the amount of the drug that is present in the supernatant will be determined. Following is an equation that will be used to calculate the EE percentage.


$$\text{EE \% =}\;\frac{\text{Total amount of Sorafenib tosylate (ST)(mg)}-\text{ Amount of Sorafenib tosylate (ST})\text{ in supernatant (mg)}}{\text{The total amount of Sorafenib tosylate (ST) (mg)}}*100$$

### 2.6 *In vitro* dissolution

A previously described dialysis method was used with minor modifications to release Sorafenib tosylate (ST) *in vitro*. A dialysis membrane bag (molecular weight cut off: 12-14 kDa) was filled with an amount equivalent to drug suspension or formulation containing 1 mg of Sorafenib tosylate (ST) diluted (1:5) in phosphate buffer and sealed. This bag was placed in a preheated 100 mL medium of simulated intestinal fluid (pH 6.8) with 0.5% T-80 in a beaker. In a thermostat shaker, the beaker was continuously shaken at 100 rpm at 37 ± 1°CC. At 5, 10, 15, 30, 30, 60, 120, 240, 480, 720, 960, and 1440 minutes, samples were withdrawn and an equal amount of dissolution media was replaced. The amount of drug in the supernatant was determined using the developed UV-UPLC method after the withdrawn samples were centrifuged for 10 minutes at 10,000 rpm. Further *in vitro* drug release kinetics studies were also analysed.

### 2.7 Cell culture

The National Centre for Cell Science in Pune, India, provided human female colorectal adenocarcinoma cell lines (SW48 Cells PTEN) (33). The cells were cultured in DMEM culture medium supplemented with 10% v/v FBS (Cytiva; India) and 1% v/v penicillin-streptomycin and kept at 37°C in a humidified incubator with 5% CO_2_.

#### 2.7.1 *In vitro* cytotoxicity

The MTT assay was utilised in order to determine whether or not the chosen formulations were cytotoxic to SW48 Cells PTEN cell lines (34). This procedure was previously described in our article. In a nutshell, 1 x 10^5^ cells were seeded into each well of a 96-well plate and left there for 24 hours. After that, the cells were treated with different concentrations (2.5–20 µg/mL) of pure sorafenib tosylate (ST), which had been pre-treated with DMSO, as well as drug-loaded formulations (ST-SLN1, ST-SLN3, and ST-NLC1, ST-NLC3). After an initial incubation period of 48 hours, 10µL of an MTT solution containing 5 mg/mL was added to each well, and the plates were then kept in the dark at 37°CC for another 4 hours. After that, the formazan product was solubilized with acidified isopropanol, and the absorbance was measured using a Revolutionary multi-mode Microplate Reader at a wavelength of 570 nm (BMG LABTECH, USA). In order to calculate the IC50, the dose-response curves were utilised (concentration required to inhibit cell growth by 50%). The following equation was utilised in order to determine the viability of the cells:


$$Cell\;Viability(\%)=\frac{\text{Optical density of the treated sample}}{\text{optical density of the untreated sample}}\times 100\%$$

#### 2.7.2 Flow cytometric analysis of cells apoptosis

Apoptosis is a type of cell death where a cell dies after going through a series of steps at the molecular level. This is one way that the body gets rid of cells it doesn’t need or that aren’t working right. Apoptosis is one way to get rid of cells that have changed in ways that could be harmful. If a cell’s apoptosis function isn’t working right, the cell can grow and divide out of control, which can lead to a tumour. Loss of control over apoptosis lets cancer cells live longer and gives them more time to collect mutations that can make them more aggressive as a tumour grows, encourage angiogenesis, make cell division less controlled, and stop differentiation.The important parts of apoptosis are: the cell shrinks, the cell breaks up, the cytoskeleton breaks down, the nuclear envelope falls apart, and the cell releases apoptotic bodies. In some ways, apoptosis also helps stop cancer. When cells are exposed to cytotoxic compounds, they may die by necrosis (uncontrolled cell death), apoptosis (programmed cell death), autophagy, or they may stop actively growing and dividing to stop cell proliferation.

To determine the percentage of cells that had undergone apoptosis, a flow cytometric analysis was performed using an Annexin-V/FITC/PI staining Kit (Sigma, USA) in accordance with the etiquettes provided by the manufacturer. In a nutshell, A549 cells were seeded in a 12-well plate at a density of 1 x 10^5^ cells per well (35). After an overnight incubation, the cells were treated with the IC_50_ concentration of pure sorafenib tosylate (ST), which was 4.5 µg/ml, as well as the equivalent concentration of ST-SLN1, ST-SLN3, and ST-NLC1, ST-NLC3. Following an incubation period of 48 hours, both treated and untreated cells were collected, washed with cold PBS (1x), and then resuspended in 100µL of binding buffer (1x) together with FITC Annexin V (5 L) and PI (5µL) (36). After incubating the samples for 20 minutes in the dark, 400µL of binding buffer was added, and then a ZE5 Cell Analyzer was used to examine the results (Bio-Rad, USA).

#### 2.7.3 Effect of Sorafenib tosylate on cell migration by using i*In vitro* scratch assay

SW48 cells PTEN cell lines were seeded (5x10^4^) in a six-well plate, and the monolayer of 80% confluent cells was treated for 48 hours with 1) Control, 2) Sorafenib tysolate, 3) ST-NLC, 4) TPGS-NLC, 5) ST-TPGS-NLC. The serum concentration in the growth media decreased to minimize the proliferation of cells so that it does not interfere with the measurement of cell migration. After 48 hours of incubation, the drug-containing solution was replaced, and a sterile p200 pipette tip was used to scrape the plates, which were then washed two times with Phosphates Buffer Saline (PBS) to kill any floating cells before being incubated in absolute **Dulbecco’s Modified Eagle Medium** (DMEM) with 5% FBS. To eliminate any potential variance caused by the disparity in scratch diameter, care was taken to produce scratches of roughly equal size in the test and control cells. Using an inverted phase microscope, we tracked cell migration by taking photographs at 0, 24, and 48 hours (Olympus, CKX41). When the cells migrate into the gap (scratch), the reduction in gap area reflects cell migration which is expected in untreated (control) cells, whereas in drug-treated cells, no reduction in gap area from ‘0’ h reflects inhibition of cell migration. As a result, the impact of the drug therapy was determined by comparing the percentage of cell migration during each time to the untreated control by using the formula below.


% Cell Migration =(Gap are at 0h− Gap area at specific time interval/Gap are at 0h) *100

For normalization, the cell migration of the untreated control at 48 hours was set to 100% (i.e., no inhibition). The effects of the other treated groups were expressed with this.

#### 2.7.4 *In vivo* anti-tumour efficacy study

In the *in vivo* effectiveness study, 8–10-week-old female severe combined immunodeficiency disorder (SCID mice) were used. Mice have kept in individually ventilated cages with a pelleted food, a 12-hour/12-hour dark-light cycle, and unrestricted access to water (provided ad libitum) in a climate-controlled setting with a temperature range of 25°C ± 3°C. All animal experiments, including those at Animal House of SPTM, NMIMS, Shirpur, Maharashtra, India, were done according to the rules set by the National Institutes of Health for the care and use of animals. Committee for the Purpose of Control and Supervision of Animal Experiments on Animals (CPCSEA) rules were also followed in these areas(SPTM/2022/IAEC/Protocol No.25). SW48 Cells PTEN in (5x10^6^ cells) in 200µL of RPMI 1640 with 33% Corning Matrigel Basement Membrane Matrix were injected under the skin of mice (Sigma-Aldrich, India). As instructed by the experimental protocols, when the average size of the xenograft tumours reached 150 mm^3^, the following treatments were given to mice in four groups (n=6): saline, sorafenib tosylate (ST) (20 mg/kg), ST-NLC (20 mg/kg), and ST-TPGS-NLC (20 mg/kg) through the lateral tailvein once a week (QW). Twice a week, the size of the tumour was measured with a digital electronic calliper. Twice a week, the tumor’s size was measured with an electronic calliper. Bodyweight was checked twice weekly to determine toxicity. Tumour volume was calculated as (width^2^ length/^2^).

### 2.8 Statistical analysis

The data were statistically evaluated using Originpro software, Version 9.0. The results were compared using an independent t-test (for data with two sets) (for data with two sets). Data were expressed as mean ± SD. P-value<0.05 was used as the criterion for significance.

## 3 Results and discussion

### 3.1 Physicochemical properties of Solid Lipid Nanoparticles, Nanostructural Lipid Carrier, and Liquid Nano Emulsion

**Figure 1:**
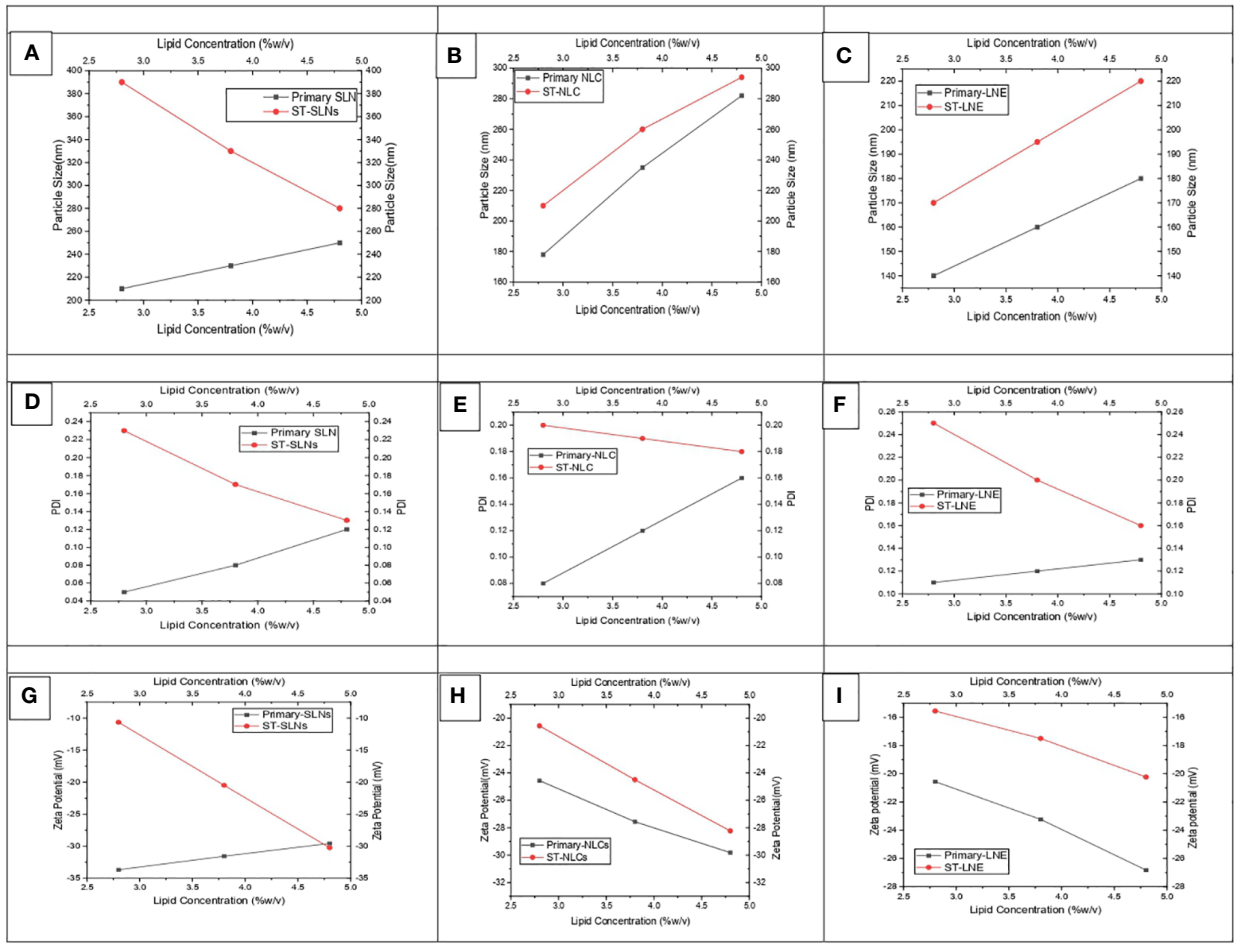
**(A-C)** particle size, **(D-F)** PDI, and **(G-I)** zeta potential values of prepared both drug-free and drug-loaded SLN, NLC, and LNE.

**Figures 1A–C** represents the particle size of primary (black colour) and ST-loaded (red colour) Solid Lipid Nanoparticles (SLN), Nanostructural Lipid Carrier (NLC), and Liquid Nano Emulsion (LNE) respectively. It was observed that increased in particles size attributes to increase in lipid content and decrease of surfactant concentration. The gained results have symmetrical orientation with forgoing formulation of different lipid-based nanoparticles. It was observed that the primary nanoparticles emulsions are having lower particle size however increase in concentration of lipids and TS could expend the core surface of the nanoparticles. The findings of this research coincide with the findings of recently published articles which was stating that, the plain lipid-based nanoparticles possessed smaller particle size in compare to drug loaded Solid Lipid Nanoparticles (SLN), Nanostructural Lipid Carrier (NLC), Liquid Nano Emulsion (LNE). According to Somayeh Vandghanooni et al. (37), solid lipid nanoparticles coated with acriflavine had a mean particle size of 106 5.7 nm. The presence of Precirol^®^ was what caused the increased particle size (37). Similar to this article, Forough Rasouliyan et al. (38) reported higher diameter of the Lawsone loaded nanoparticles (LWS-SLNs) when 450 mg Precirol^®^ utilised as solid lipids (38). The decrease in the viscosity of the lipid core as a result of sonication may be responsible for the smaller particle size of SLN that was achieved after either the complete or partial replacement of solid lipid with liquid oil in the Nanostructural Lipid Carrier (NLC) or Liquid Nano Emulsion (LNE), respectively. The melting points of Stearic acid, Stearic acid: Oleic acid (3:1), and Oleic acid were measured to be 73.1°CC, 54.8°CC, and 14°CC, respectively, in this experiment. As a result, it is anticipated that increased levels of medium viscosity will be achieved during the production of SLN. Smaller particles size of nanoparticles might be possible due to the lower melting points of the lipids. Resveratrol (RSV) is encapsulated as solid lipid nanoparticles (RSV-SLNs) in the presence of Compritol 888ATO and Myglyol as solid lipids, which causes a decrease in the melting points of the lipids, according to the research of Evren H Gokce et al. (39) However, a significant linear relationship between concentration of lipid and particle size was observed between Primary-Nanostructural Lipid Carrier (NLC) and ST-NLC & Primary-Liquid Nano Emulsion (LNE) and ST-LNE. But the particle size relationship of primary-SLN and ST-SLNs was found to be antagonistic **(** **Figure 1A** **)**; which could be because of the extensive use of Stearic acid (SA) and the resultant decrees of nature of crystallinity produced by SA while SLNs were being prepared. ST-SLN3 was found to have a low particle size despite having a higher SA concentration because of the noticeably lower drug lipid ratio. It is also possible to draw the conclusion that ST was easily diluted in the lipid, allowing SA to be packed tightly inside the SLN core. However, there are certain exceptions, such as the itraconazole-loaded solid lipid nanoparticles made by Biswaranjan Mohanty et al. (40) using stearic and palmitic acids. It was discovered that the itraconazole-encapsulated stearic acid solid lipid nanoparticles had a larger mean particle size (139-199 nm) than the itraconazole-encapsulated palmitic acid solid lipid nanoparticles (126-160 nm). Itraconazole’s crystalline structure and poor intrinsic solubility in lipids may be too responsible for the higher particle size in stearic acid-encapsulated itraconazole solid lipid nanoparticles (40). It has been suggested in several studies that the presence of lipophilic drugs within solid lipids may reduce lipid crystallinity. All-trans retinoic acid (RA) and stearylamine (STE), when combined with cholesterol (CHO) and polyoxyl 20 cetyl ether to create solid lipid nanoparticles, were shown to display little drug crystallisation within lipids, according to Gisele A. Castro et al. s research (41). Following a similar trend, Ilaria Arduino et al. (42) created solid lipid nanoparticles (SLNs) by conjugating Pt(IV) prodrugs with cetyl palmitate in PEG. In this study, Pt(IV) solubility in lipids rose along with a decline in crystallinity (42). The PDI values for all three formulations were depicted in **Figures 1D–F**. Primary nano formulation (black colour line) and ST-loaded SLNs, NLCs, and LNE showed a parallel trend. Most importantly, every formulation has a PDI value lower than 0.3, which indicates a stable and homogeneous emulsion. This implies a reproducible therapeutic outcome and a physically stable nano emulsion. The zeta potential values of Plain (black colour) and ST-loaded (red colour) SLN, NLC, and LNE are shown in **Figures 1G–I** respectively. The expectation of formulation stability depends on the nanoparticles’ zeta potential value. As a result, it is anticipated that increasing the zeta potential value, whether on the positive or negative side, will improve stability and stop particles from aggregating due to the repulsion effect. Additionally, negative nanoparticles have longer circulation times because they are less susceptible to the opsonization process. The zeta potential value of the prepared formulations was negative, which is expected to improve stability and therapeutic outcome (43).

### 3.2 Particles morphology

**Figure 2:**
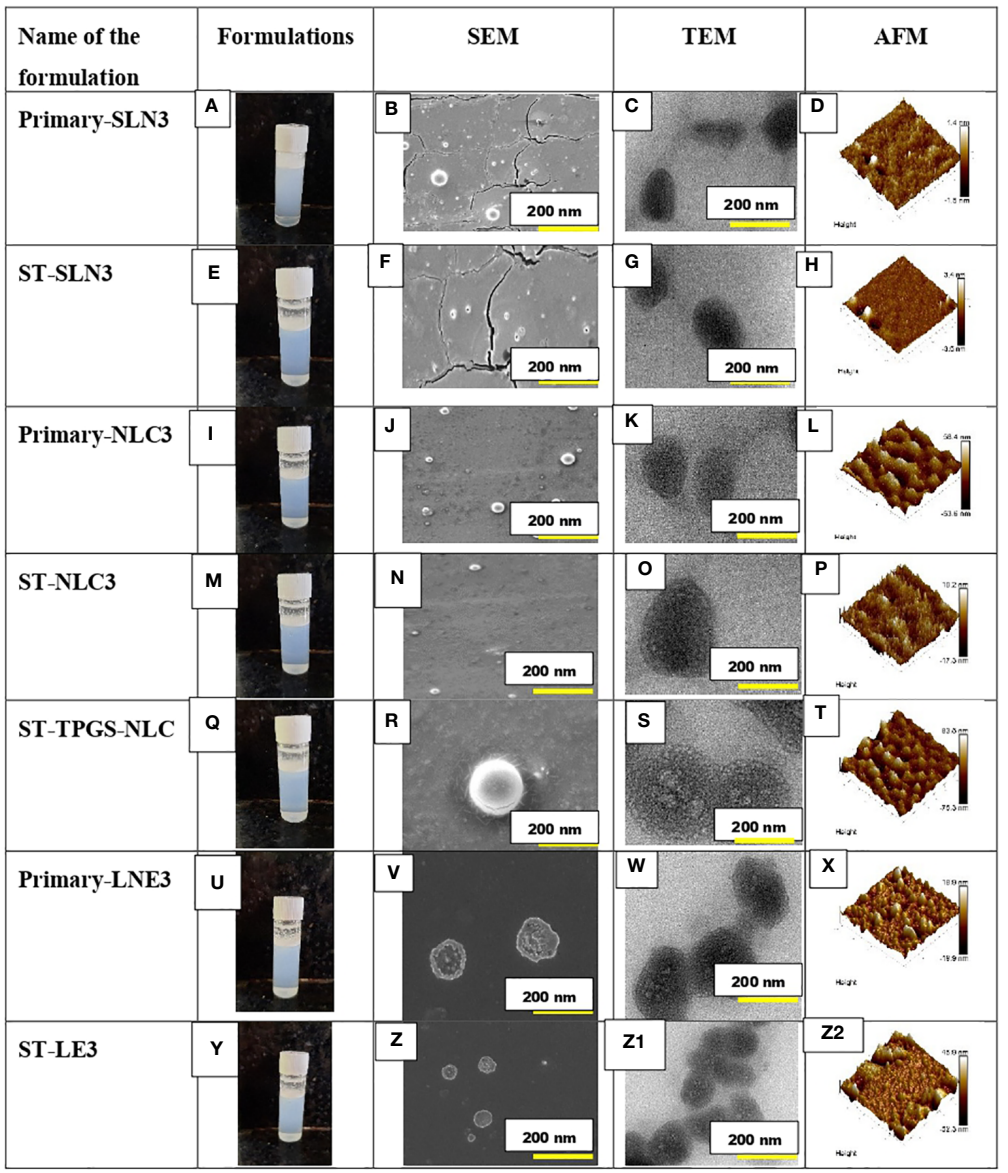
**(A-D)** represents the outline, SEM, TEM and AFM image of Primary-SLN3. **(E-H)** represents the outline, SEM, TEM and AFM image of ST-SLN3. **(I-L)** represents the outline, SEM, TEM and AFM image of Primary-NLC3. **(M-P)** represents the outline, SEM, TEM and AFM image of ST-NLC3. **(Q-T)** represents the outline, SEM, TEM and AFM image of ST-TPGS-NLC. **(U-X)** represents the outline, SEM, TEM and AFM image of Primary-LNE3. **(Y-Z2)** represents the outline, SEM, TEM and AFM image of ST-LE3.

Prior to mooring, it is essential to characterise the established morphological profile of ST-loaded SLNs, NLCs, and LNE. **Figures 2A–Y** depicts the formation of Primary-SLN3, ST-SLN3, Primary-NLC3, ST-NLC3, ST-TPGS-NLC, Primary-LNE3 and ST-LE3. In order to comprehend the morphological behaviour of ST encapsulated SLNs, NLCs, and LNE, the SEM analysis was carried out **(Figures 2B–Z)**. The SEM instrument’s electrical bombardment may cause a thermal fluctuation that causes liquids to evaporate from inside nanoparticles and causes smooth, regular, spherical-shaped lipid nanoparticles to tender. With the help of TEM, it is easy to identify the encapsulation of ST within lipidic nanoparticles. While TEM operation, samples were placed on 300-mesh coper coated gride. The spherical Primary-SLN3, ST-SLN3, Primary-NLC3, ST-NLC3, ST-TPGS-NLC, Primary-LNE3 and ST-LE3 can be observed in **Figures 2–Z1.** The TPGS conjugation in ST-TPGS-NLC can easily identify in **Figure 2R, S**. It should be noted that particles lose moisture during TEM measurements. According to Yupei Wu et al. (44), paclitaxel-loaded derivative nanoparticles based on d-α-tocopherol polyethylene glycol succinate were created, and it was also claimed that these particles had morphologically (based on TEM data) smaller particle diameters than those found in DSL studies (44). In similar to our findings according to Matte Kasi Viswanadh et al. (45) Novel redox-sensitive thiolated TPGS based nanoparticles has demonstrated shirked particle shape in TEM images (45). Additionally, as previously mentioned, the likelihood of aggregated particles while measuring in Delsa Nano C instruments was extremely high due to the polymeric nanosuspension having been sonicated for 15 min prior to size measurement. A three-dimensional analysis of the surface morphology was achieved using semi-contact atomic force microscopy (AFM), which was carried out under a high vacuum. 3D images and Nova navigation software were used to examine the height, Average skewness, roughness (nm), and kurtosis of Primary-SLN3, ST-SLN3, Primary-NLC3, ST-TPGS-NLC, Primary-LNE3, and ST-LE3 nanoparticles **Figures 2 (D-Z2)**. Because TPGS has been conjugated to the surface of ST-TPGS-NLC, it has a smooth texture and a significantly higher surface height than all other lipid nanoparticles. Average skewness, roughness (nm), and kurtosis values for the ST-TPGS-NLC were 0.413 ± 0.23, 86.4 ± 4.20 nm, and 0.521 ± 0.050, respectively.

### 3.3 DSC

**Figure 3:**
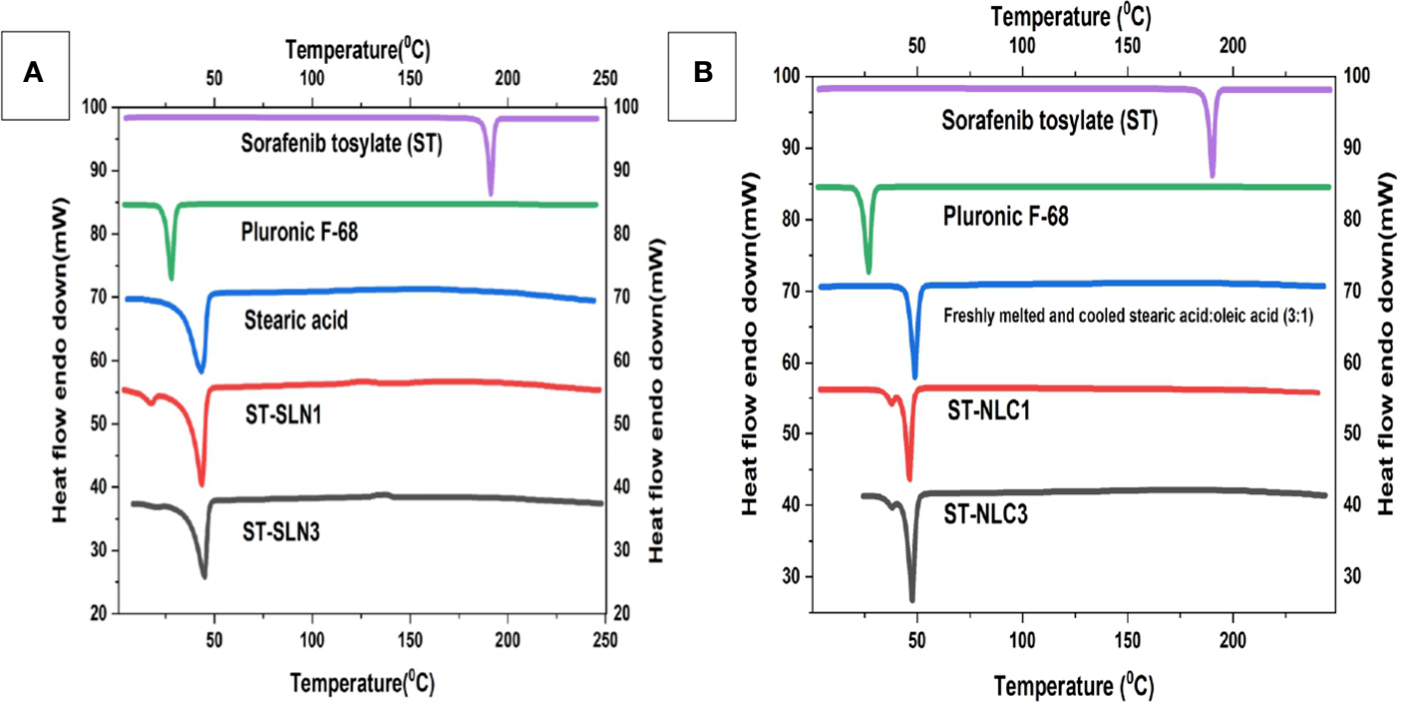
**(A)** Differential scanning calorimetry thermogram of Sorafenib Tosylate (ST), Pluronic F-68, freshly melted and cooled Stearic acid (SA), ST-SLN1, ST-SLN3. **(B)** Differential scanning calorimetry thermogram of freshly melted and cooled Stearic acid (SA): oleic acid (3:1), ST-NLC1, and ST-NLC3.

Stearic acid (SA), Pluronic F-68, newly melted then cooled SA, ST-SLN1, and ST-SLN3 are all compared on the differential scanning calorimeter graph in **Figure 3A**. Additionally, **Figure 3B** shows the DSC graphs of ST, freshly melted Pluronic F-68, freshly melted and cooled SA: oleic acid (3:1), ST-NLC1, and ST-NLC3. The melting temperatures of ST and Pluronic F-68 were determined to be 200.8°CC and 25.5°CC, respectively, which are nearly identical to earlier estimates. Duy Hieu Truong et al. (46) research shows a sorafenib DSC endothermic peak at 238°C, which is consistent with our findings. However, in the same article, they reported the melting point of the tosylate salt form and one small endothermic peak at about 200°C, which may be indicative of the melting point of the sorafenib base form (46). On freshly melted and then later cooled SA, SA: Oleic acid (3:1) mixes, a DSC scan was performed to examine the impact of the process on the melting point. It was discovered that the melting points were 49.2°CC and 50.3°CC, respectively. The melting endotherm of stearic acid was determined to be 65°CC in Timothy M. Amis et al. (47) findings on progesterone-loaded stearic acid solid lipid nanoparticles; which nearly match our findings (47). The modest shifting and broadening of the SA peak may be due to the presence of oleic acid, which reduces the melting point and crystallinity of Stearic acid (SA). This is due to the fact that oleic acid reduces SA’s crystallinity. The results of various researchers who discovered that a range of liquid oils lowered the melting point of solid lipid, were compatible with this. The melting points of formulations utilising ST-SLN were extremely near stearic acid’s melting point (SA). At 48.4°CC for the ST-SLN1 formulation and 47.1°CC for the ST-SLN3 formulation. While ST-NLC3 had a melting point of 50.2°CC, ST-NLC1 had a melting point of 50.1°CC. Similar findings were found using ST-NLC formulations. However, neither the ST-SLN nor the ST-NLC had a melting point for ST, which would suggest either that the drug is present in an amorphous condition or that it is evenly dispersed throughout the lipid core. According to the findings, several researchers showed that the incorporation of the lipophilic drug into SLN and NLC caused the elimination of the drug peak to take place. This result showed that the drug was present in the lipid phase either in an amorphous condition or in a homogeneous distribution. In similar fashion, Adaeze L. Onugwu et al. (48) created ciprofloxacin (CIP) solid lipid nanoparticles using chitosan and poly(2-ethyl-2-oxazoline). The optimized formulations displayed endothermic peaks at 73.95°C and 73.90°C, but the CIP peak at 73.95°C was not present, indicating that CIP had been fully dissolved and encapsulated in the lipids (48).

### 3.4 PXRD

**Figure 4:**
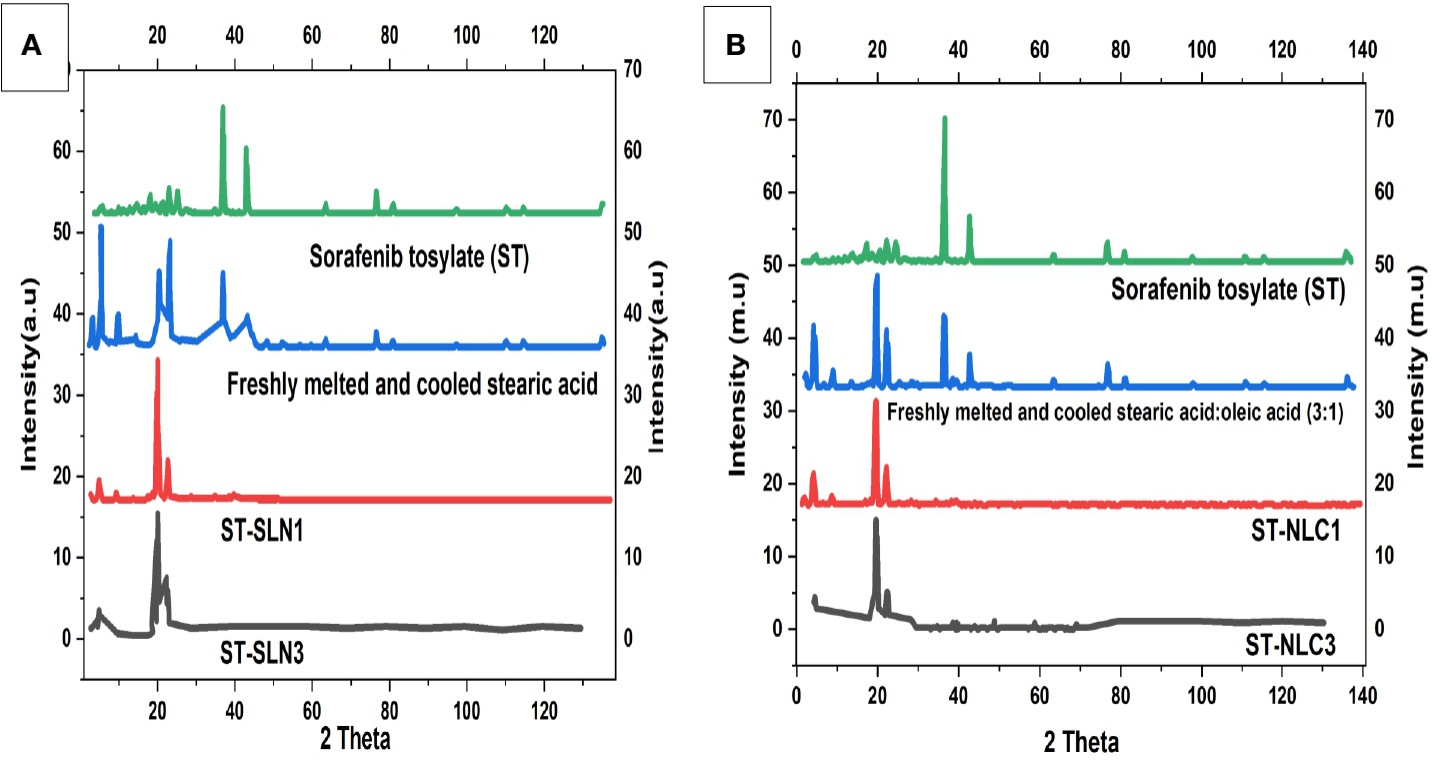
**(A)**. Powder X-Ray Diffraction (PXRD) diffractogram of Sorafenib Tosylate (ST), freshly melted and cooled Stearic Acid (SA), ST-SLN1, ST-SLN3 **(B)**. **(A)**. Powder X-Ray Diffraction (PXRD) diffractogram of freshly melted and cooled Stearic Acid (SA): SA (3:1), ST-NLC1, and ST-NLC3.

The diffraction patterns of ST, freshly melted and cooled SA, ST-SLN1, and ST-SLN3 are displayed in **Figure 4A**. The DSC graph of ST, freshly melted and cooled SA, ST-NLC1, and ST-NLC3 is also displayed in **Figure 4B**. ST’s PXRD graph displays multiple moderately intense peaks at 20.4°C, 23.8°C, 27.2°C, and 76.3°C in addition to high intensity peaks at 39.1°C and 37.4°C. Furthermore, high-intensity diffraction peaks at 6.7°C, 12.8°C, 23.7°C, 24.3°C, 38.1°C, and 42.3°C were visible in freshly melted and cooled SA. Freshly melted and cooled SA: SA (3:1) had a different diffraction pattern from SA in that the peaks at 22.7°C, 26.3°C, 39.2°C and 46.3°C were less prominent. Last but not least, the PXRD pattern of the ST-SLN and ST-NLC formulations reveals a sharp reduction in the peaks at 7.1°C and 12.4°C as well as the disappearance of the two dominant SA and ST peaks at 39.4°C and 42.8°C. The SA diffraction pattern displayed numerous, intense peaks, which indicated a high level of crystallinity. Moreover, the fluidization effect created by liquid oil is blamed for the decrease in SA crystallinity following the incorporation of OA. The formulations with the highest and lowest solid lipid: drug ratios also underwent PXRD. The disappearance of the dominant peak of ST in the PXRD pattern indicated the presence of the drug in an amorphous state. Additionally, the disorientation of crystals packing in the presence of ST may be responsible for the decrease in SA peaks. Phase 1 of the preparation of the stearic acid nanocomposite produced a V-type polymorph with both short- and long-ranged crystalline structure, according to Hye-Young Shin et al. (49).

### 3.5 Stability of prepared formulations

**Figure 5:**
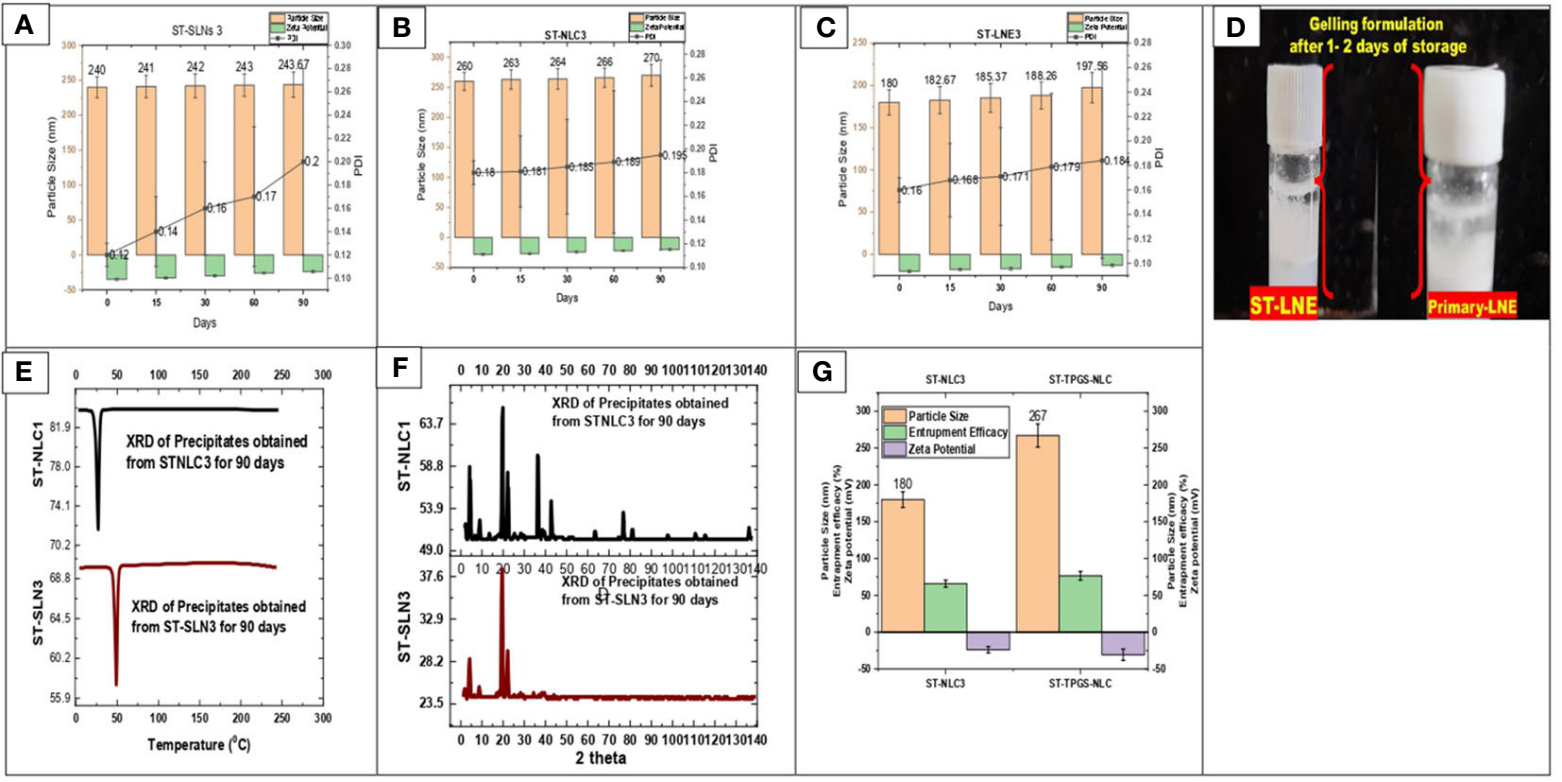
**(A)** Particle size, Zeta potential, PDI of ST-SLNs 3 **(B)** Particle size, Zeta potential, PDI of ST-NLC 3 **(C)** Particle size, Zeta potential, PDI of ST-LNE3 **(D)** Formation of Gel after 1-2 days storage of ST-LEN and Primary LEN **(E)** DSC **(F)** PXRD of ST-SLN and ST-NLC formulations. **(G)** Particle size, entrapment efficiency, zeta potential of ST-NLC3 and ST-TPGS-NLC.

During the course of the stability study, **Figure 5** depicted the physicochemical characteristics of the ST-SLNs, ST-NLC and ST-LEN formulations. For the purpose of the stability study, each formulation was placed in a cold storage unit for a period of three months before being analysed physiochemically. After a storage period of one or two days, the Primary-LNE and ST-LNE formulations both exhibited gelling behaviour **(** **Figure 5D** **)**. In spite of the fact that Plain-SLN demonstrated a high degree of stability, ST-SLN was unstable, and particles aggregation was observed after three months had passed. This phenomenon might be explained by the movement of the drug away from the lipid core and toward the nanoparticles’ coating. As a consequence of this, there was an appreciable change in the surface properties, and particle aggregation was observed. As a result, aggregated particles that had settled to the bottom of the bottle in both the high and low drug: lipid ratio conditions (referred to as ST-SLN1 and ST-SLN3, respectively) were collected. In order to characterise them, DSC and PXRD analysis were carried out, as shown in **Figures 5E, F**. The Thermos-scanning can reveal additional endothermic peaks between 50°CC and 45°CC, and these peaks could be attributed to ST. In the case of the ST-SLN1 precipitate, these peaks are more pronounced than in the case of the ST-SLN3 precipitate. In addition to this, crystalline peaks of ST were observed by PXRD when applied to aggregated particles. In addition to this, the PXRD diffraction pattern exhibits peaks at 20.10°C and 21.40°C, and in the case of the ST-SLN1 precipitate, these peaks are more pronounced. When all of these factors are considered, explain how ST progressing from core to coated resulted in particles aggregation. When it came to ST-NLC, the formulations that were prepared were stable, and the aggregation of particles was not something that was seen. The solubilization of ST in liquid oil may be responsible for this phenomenon (oleic acid). Consequently, using liquid oil in addition to solid lipids ensures the formulation’s stability. The presence of oleic acid in a greater quantity, which changes the surface properties of nanoparticles, could be responsible for the instability of ST-LNE. It’s possible that this is what’s causing the instability and the observed aggregation of particles. **Table 2** provides a summary of the prepared lipid-based nanoparticles’ physicochemical properties as well as their stability (SLN, NLC, and LNE).

**Table 2:** Stability and physicochemical properties of the prepared nanoscale lipid-based nanoparticles.

Formulation type	SLN		NLC		LEN	
	Primary-SLN	ST-SLN	Primary-NLC	ST-NLC	Primary-LEN	ST-LE
Lipid core composition	Solid lipid		Solid lipid beside liquid oil		Liquid oil	
Particle size (nm)	210-241	258-298	186-220	221-242	148-200	165-236
PDI	0.072-0.160	0.149-0.288	0.181-0.201	0.125-0.191	0.154-0.192	0.160-0.261
Zeta potential (mV)	(-25) to (-21)	(-27) to (-21)	(-30) to (-24)	(-31) to (-24)	(-28) to (-27)	(-26) to (-16)
Stability	3 months stability observed	1 month stability observed	3 months stability observed		Unstable and forms gels within 1-2 days	
Decision	——	Omitted	——	Designated	——	Omitted

### 3.6 Physicochemical properties of ST-TPGS-NLC formulation

The effect of TPGS on the physicochemical properties of ST-NLC is depicted in **Figure 5G**. After TPGS incorporation, the particle size of ST-NLC increased from 180 ± 10.23 nm to 267 ± 10.56 nm. In addition, the zeta potential value was increased from –25,4 mV to –31,4 mV without any discernible change. Lastly, neither formulation demonstrated a significant difference in entrapment efficiency (64.67% to 74.35%), indicating that the two formulations were comparable. The coating effect produced by the PEG component of TPGS may account for the enlargement of the NLC size. Moreover, the incorporation of TPGS did not significantly alter the EE of loaded drugs.

### 3.7 *In vitro* release

**Figure 6:**
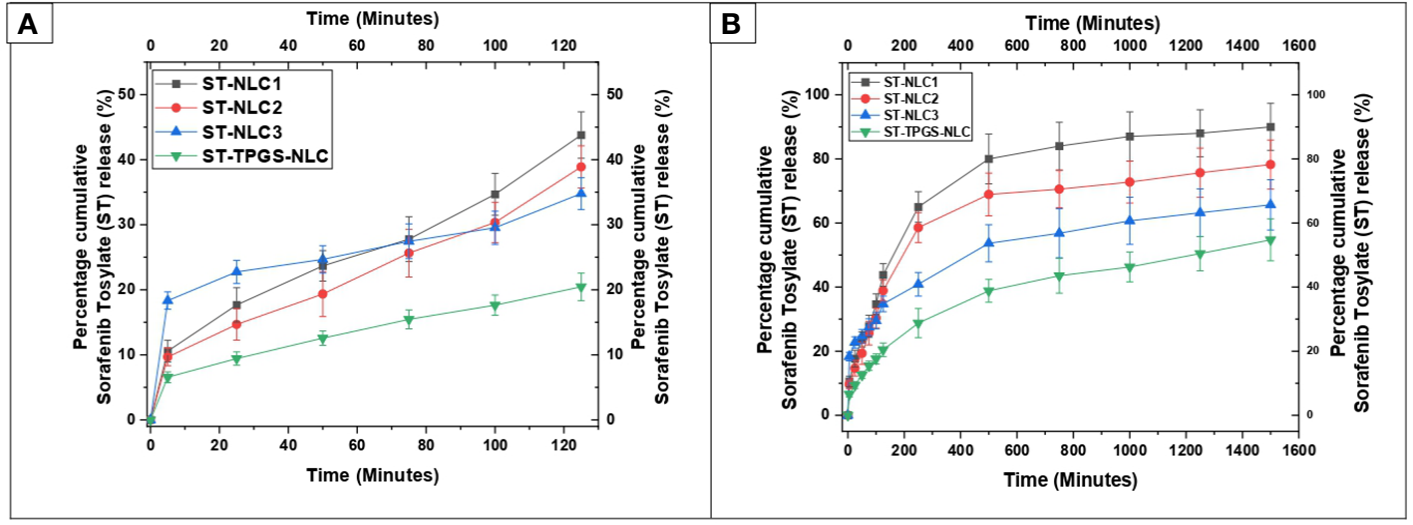
*In vitro* drug release profile of ST-NLC1-3 and ST-TPGS-NLC in phosphate buffer containing 0.5% Polyoxyethylene sorbitan monooleate. **(A)**. Percentage cumulative sorafenib tosylate (ST) release profile from nanoparticles up to 120 minutes **(B)**. Percentage cumulative sorafenib tosylate (ST) release profile from nanoparticles up to 1600 minutes. The data were expressed as the mean ± SD, with N=3.

The dissolution profiles of ST-NLC1, ST-NLC2, ST-NLC3, and ST-TPGS-NLC are displayed in **Figure 6**. The figure makes it abundantly clear that each formulation exhibited zero-order behaviour and exhibited burst drug release for a maximum of 120 minutes **(** **Figure 6A** **)**. In addition, sustained drug release was observed for up to eight to twelve hours, after which there was no notable drug release until the experiment was finished **(** **Figure 6B** **)**. When compared to the other formulations of ST-NLC, ST-NLC1 demonstrated a faster drug release than the other formulations (ST-NLC2, ST-NLC3). When compared to other formulations, this could be attributed to the fact that the drug was able to be solubilized using a relatively small amount of lipid. Therefore, the fact that the drug stays contained within the lipid nanoparticles until it is able to pass through the intestinal membrane guarantees that the lymphatic system will receive the therapeutic agent that was administered. The ST-NLC3 formulation was chosen as the best option, and it was put through a dissolution test so that the effect of TPGS on drug release could be investigated. The data presented in the figure make it abundantly clear that TPGS did not demonstrate any appreciable variation in drug release. Many researchers came to the conclusion, in line with the findings that were obtained (50, 51), that TPGS coated ST-NLC produces a more sustained drug release when compared to uncoated NLC. This could be attributed to the increased thickness of the diffusion layer, which was found to be present in the TPGS coated NLC. Additionally, it was found in another study that loaded drug was released more quickly from unmodified NLC compared to TPGS coated NLC and that was a significant finding of this research.

The drug release kinetics of the formulations were determined by fitting the *in vitro* drug release data into different kinetic models to find the best fit. The kinetic model was chosen based on which one had the highest R^2^ value and the lowest regression sum of squares (SSR). The *in vitro* drug release of Sorafenib tosylate (ST) nanoparticles in phosphate buffer (pH 6.8) showed that the nanoparticles are matrix type. The Sorafenib tosylate (ST) nanostructured lipid carriers (NLC) followed the Korsmeyer-Peppas model, which showed that diffusion was the main factor in drug release. The Korsemeyer-Peppas n-value was less than 0.4, proving that the drug release from the Sorafenib tosylate (ST) nanostructured lipid carriers (NLC) followed Fick’s law of diffusion **(** **Table 3** **)**.

**Table 3:** Drug release kinetics of Sorafenib tosylate (ST) nanostructured lipid carriers (NLC) in pH 6.8 phosphate buffer.

		ST-NLC1	ST-NLC2	ST-NLC3	ST-TPGS-NLC
Zero	R^2^	0.90	0.90	0.85	0.99
	Ko	6.66	6.68	8.58	8.70
	SS	180.33	243.44	551.40	262.0
First	R^2^	0.93	0.94	0.95	0.98
	K1	0.08	0.08	0.12	0.15
	SS	78.32	102.31	176.22	63.10
Higuchi	R^2^	0.90	0.92	0.92	0.90
	K_H_	15.42	15.47	20.25	20.35
	SS	202.45	157.22	201.20	301.21
Korsemey er Peppas	R^2^	0.98	0.97	0.98	0.99
	K_KP_	10.63	11.81	16.72	13.72
	SS	70.62	71.60	120.08	72.04
Hixson Crowell	R^2^	0.95	0.90	0.91	0.97
	K_HC_	0.02	0.02	0.02	0.03
	SS	102.40	147.20	270.20	94.21

### 3.8 *In vitro* cytotoxicity

**Figure 7:**
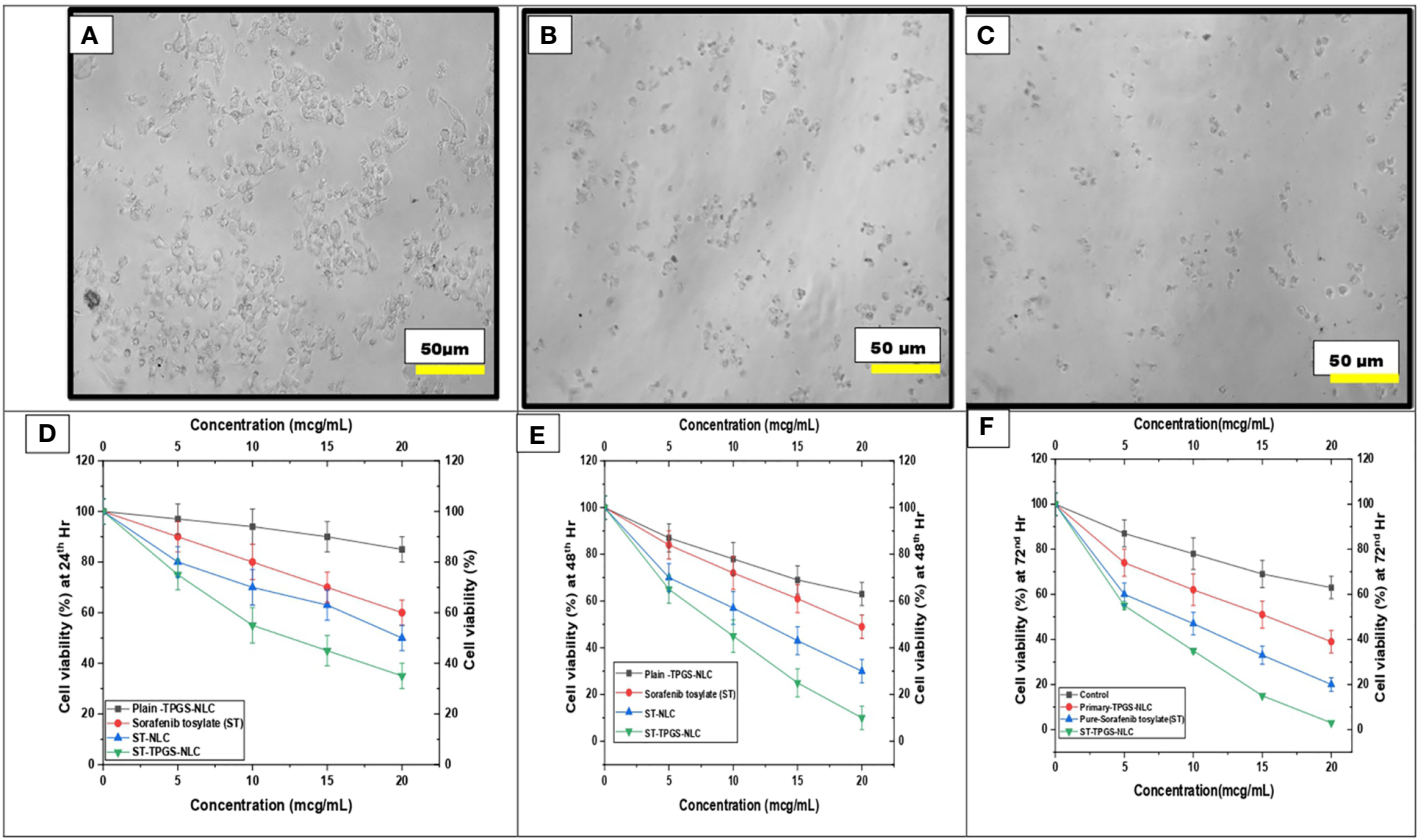
Effect of pure ST, Plain/primary-TPGS-NLC, ST-NLC, and ST-TPGS-NLC on cell viability of human female colorectal adenocarcinoma cell lines (SW48 Cells PTEN) using MTT assay treated with different concentrations (0, 5, 10, 15 and 20 μg/mL) after **(A, D)** 24 hr and **(B, E)** 48 h. and **(C, F)** 72 h Data were expressed as the mean ± SD, N = 3.

It has been hypothesised that the nanoscale pharmaceutical formulations will increase the cytotoxicity of the chemotherapeutic agents. In this study, the optimised ST-NLC3 and ST-TPGS-NLC were chosen to achieve the *in vitro* cytotoxicity study due to their ability to retain the drug, which may increase their susceptibility to lymphatic delivery. The *in vitro* cytotoxicity study was carried out to determine whether or not the drug was toxic to cells *in vitro*. An MTT assay was used to investigate the effects of pure ST, Primary-TPGS-NLC, ST-NLC, and ST-TPGS-NLC on the growth of human female colorectal adenocarcinoma cell lines (SW48 Cells PTEN). These cell lines were used to study the inhibitory effects of ST. **Figures 7A–F** showed the impact of formulations at four different concentrations (0,5,10,15, and 20 µg/mL) on the samples’ cell viability after 24, 48 and 72 hours of incubation. The toxicity of drug-free formulation for Primary-TPGS-NLC was investigated by incubating it in an equivalent volume to ST-TPGS-NLC at each concentration. The growth of human female colorectal adenocarcinoma cell lines (SW48 Cells PTEN) was inhibited by all formulations in a concentration-dependent manner (52). The outcomes showed that after being incubated for 24 hours with Primary-TPGS-NLC at a higher concentration (20.0 µg/ml), more than 85% of the cell was able to survive. Additionally, after being incubated for 48 hours, Primary-TPGS-NLC displayed significantly less cytotoxic activity than other formulations. Furthermore, after being incubated for 24 hours, ST-TPGS-NLC was able to produce a significant amount of cytotoxic activity at all concentrations except 20 µg/ml. After 48 hours of incubation, however, there is no discernible difference in the cytotoxic activity of ST-TPGS-NLC, Pure ST, and ST-NLC. The IC50 for Pure ST, ST-NLC, and ST-TPGS-NLC at 24, 48 and 72 hours was also displayed in **Table 4**. Following a 24-hour incubation period, the respective IC50 values for pure ST, ST-NLC, and ST-TPGS-NLC were 14.82 µg/mL, 17.19 µg/mL, and 8.56 µg/mL However, after 48 hours of incubation, the IC50 dropped to 5.33 µg/mL, 6.14 µg/mL, and 4.03 µg/mL, and 72 hours of incubation, the IC50 dropped to 3.45 µg/mL, 2.56 µg/mL and 1.56 µg/mL respectively. It should be noted that at both incubation times, the IC50 for Primary-TPGS-NLC cannot be calculated. When compared to pure ST, it was discovered that ST-TPGS-NLC produced significantly more cytotoxic activity. In contrast to the ST-NLC formulation, pure ST was able to produce pronounced cytotoxic activity. There is a possibility that the presence of TPGS in ST-TPGS-NLC is responsible for its cytotoxic activity. TPGS is a type of nonionic surfactant that is composed of PEG polymer that has been covalently connected to vitamin E. The first one decreases the amount of particle clearance, while the second one improves the amount of permeation and uptake that occurs through vitamin E receptors. After an incubation period of 72 hours, there was no discernible difference in the cytotoxicity of the different formulations that were tested. This could be as a result of the formulations tested only receiving insufficient incubation time to produce cytotoxic activity (53). The findings showed that NLC had a greater ability than any other nanoparticle to increase cellular uptake, which could be attributed to its propensity for adhesion to lipid surfaces. Additionally, it was found in many research findings that drug-loaded TPGS-NLC had lower IC50 values than drug-free cytotoxic drugs and drug-loaded NLC. Additionally, a confocal microscope was used to conduct a study on cellular uptake, and it was discovered that drug-loaded TPGS-NLC has a higher intensity than drug-loaded NLC. In relation to the findings of our study, Sarinya Palakhachane et al. (54) examined the cytotoxicity of sorafenib analogues in HepG2 and Huh7 human hepatocellular carcinoma (HCC) cell lines (54). According to Yue Hu et al. (2021) study, sorafenib-surface decorated iRGD nanoparticles (Sora-NPs and iRED@Sora-NPs) exhibit excellent *in vitro* cytotoxicity against hepatocellular cancer cells (BEL-7402 and Huh-7) and exhibit low toxicity in MTT assays (55)

**Table 4:** IC_50_ of pure ST, ST-NLC, and ST-TPGS-NLC on cell viability of SW48 Cells PTEN cell line using MTT assay after 24^th^ hr and 48^th^ hr.

Duration	Pure ST (µg/mL)	ST-NLC (µg/mL)	ST-TPGS-NLC (µg/mL)
24 hr	14.82	17.19	8.56
48 hr	5.33	6.14	4.03
72 hr	3.45	2.56	1.56

### 3.9 Apoptotic study

**Figure 8:**
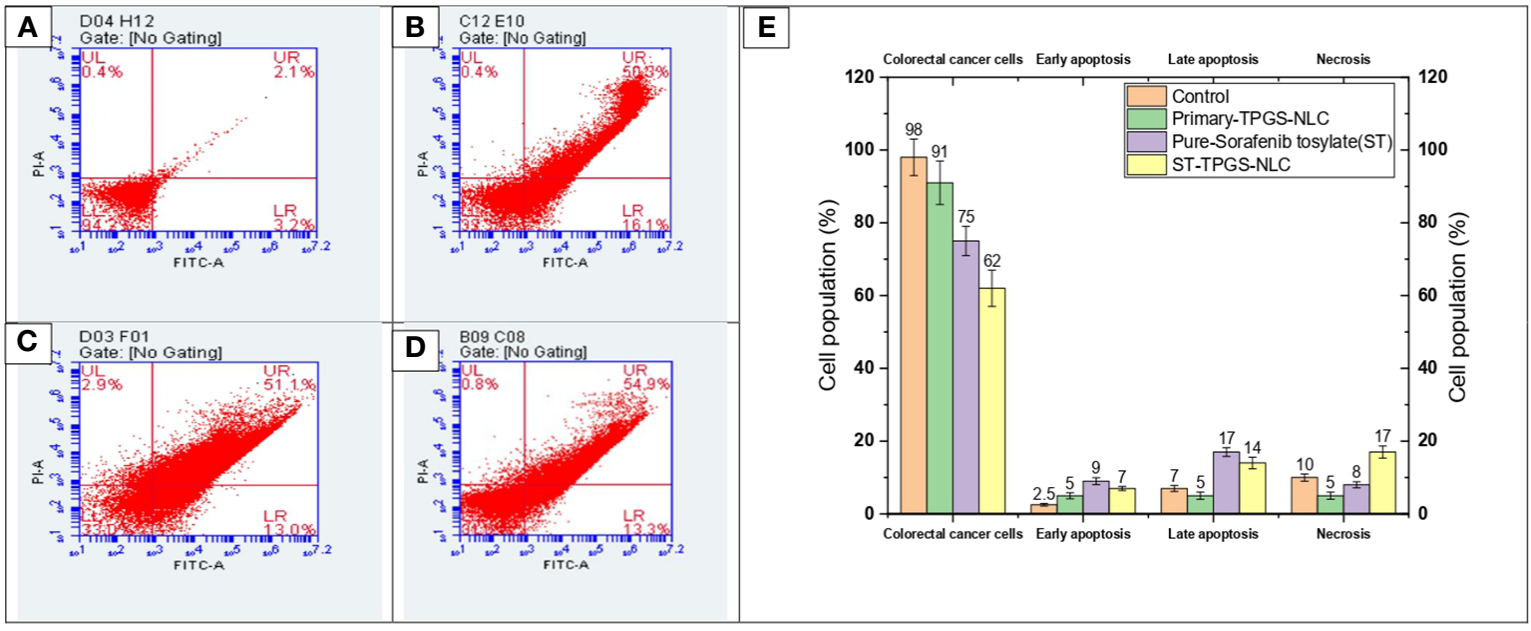
Flow cytometric analysis of SW48 Cells PTEN cell line treated with **(A)** Control, **(B)** Primary-TPGS-NLC, **(C)** pure ST, and **(D)** ST-TPGS-NLC at 12.15 μg/mL concentration. (UL, UR, LR, and LL) Necrotic, late apoptotic, early apoptotic, and viable cells are shown in the upper left quadrant, upper right quadrant, lower right quadrant, and lower left quadrant, respectively. **(E)** Bar chart shows the percentage of live, early apoptosis, late apoptosis, and necrotic cells that were treated with control, Primary-TPGS-NLCs, Pure-Sorafenib tosylate (ST), and ST-TPGS-SLN. Data were expressed as the mean ± SD, N = 3.

A complicated physiological process known as apoptosis is responsible for the elimination and removal of cells that are not wanted in the body (56–58). As a result, this study is being carried out in order to classify and evaluate the population of cells after said cells have been treated with chemotherapeutic agents. In the current investigation, human female colorectal adenocarcinoma cell lines (SW48 Cells PTEN) were subjected to a 24-hour treatment with pure ST, Plain-TPGS-NLC, ST-NLC, and ST-TPGS-NLC. The cells were then stained with a double stain of annexin V and propidium iodide (PI). Based on the results of the MTT test, the length of time spent in incubation was chosen so that a comparison could be made between pure ST and ST-TPGS-NLC. The cells were treated with an IC50-equivalent concentration of pure ST at a concentration of 12.45 µg/mL from each of the formulations. As can be seen in **Figure 8**, exposure to pure ST at a concentration of 13.47 µg/mL caused an increase in the early apoptotic cell population (15.31 ± 2.41%), the late apoptotic cell population (13.17 ± 3.02%), and the necrotic cell population (6.08 ± 1.80%), in comparison to the population of untreated cells (3.10 ± 1.41%, 4.17 ± 0.45%, and 3.98 ± 0.23%. After treating the cells with pure ST or ST-TPGS-SLN, respectively, we found that the viability of the cells significantly decreased, falling from 71.8 ± 4.8% to 62.4 ± 2.6%. This finding is quite interesting. In addition to this, the percentage of early apoptotic cells, late apoptotic cells, and necrotic cells that were present in the population after treatment with ST-TPGS-NLC was 9.3 ± 0.70%, 13.2 ± 0.82%, and 20.4 0.8% respectively. In clinical practise, the traditional administration of ST produced unsatisfactory therapeutic results when the drug was given in lower concentrations. This is because the drug is not distributed very widely, which makes it vulnerable to drug-resistant mechanisms like efflux transporters. Patients are given extremely high doses of chemotherapeutic agents in order to accomplish this goal, which results in a high level of systemic toxicity. In light of this, increasing the cytotoxic effect of the chemotherapeutic agent that is being administered not only results in fewer adverse effects, but also reduces the necessary therapeutic response. In addition to this, the encapsulation of therapeutic agents inside of an appropriate lipoprotein mimic carrier. Because of this, ST is prevented from binding to plasma proteins, which results in an increase in the proportion of free drug available for biodistribution. As a result, the newly developed ST-TPGS-NLC enhances the cytotoxic activity of ST at lower concentrations while achieving a highly desirable outcome in terms of biodistribution. Because of this, a cytotoxic agent was brought into the cell from the outside rather than the inside. Therefore, one could reach the conclusion that cytotoxic activity is produced by ST-TPGS-NLC through the mechanism described above. This not only improves the efficacy of the therapeutic outcomes, but it also lowers the toxicity of ST by reducing the amount of the drug that is required for treatment. When everything is considered together, it is anticipated that TPGS engineered nanoscale lipid-based LDDS will increase the therapeutic outcomes of ST in the treatment of Colorectal cancer (59). Similar results were previously reported in Mushfiq Akanda et al. (60) work, where the researcher reported 50 µg/mL Tf-CRC-SLN exhibited significant increase in late apoptotic/early necrotic cell population only (p<0.05) compared to the curcumin (CRC)loaded SLN during formulation development and *in vivo* studies on prostate cancer through bio conjugated solid lipid nanoparticles (60). In a similar vein, Han Li et al. (2022) article suggests that the combination of sorafenib and ursolic acid may involve the induction of Mcl-1-related apoptosis and SLC7A11-dependent ferroptosis (61). Their research’s conclusions present a brand-new, highly effective therapeutic approach for the treatment of tumours. By avoiding unneeded inflammation in the healthy cell compartments and reducing side effects in patients during treatments, the use of such a targeted drug delivery system may be able to reduce the highly necrotic behaviour of anticancer molecules.

### 3.10 Effects of Sorafenib tysolate nano formulation on colon cancer growth and angiogenesis in nude mice

**Figure 9:**
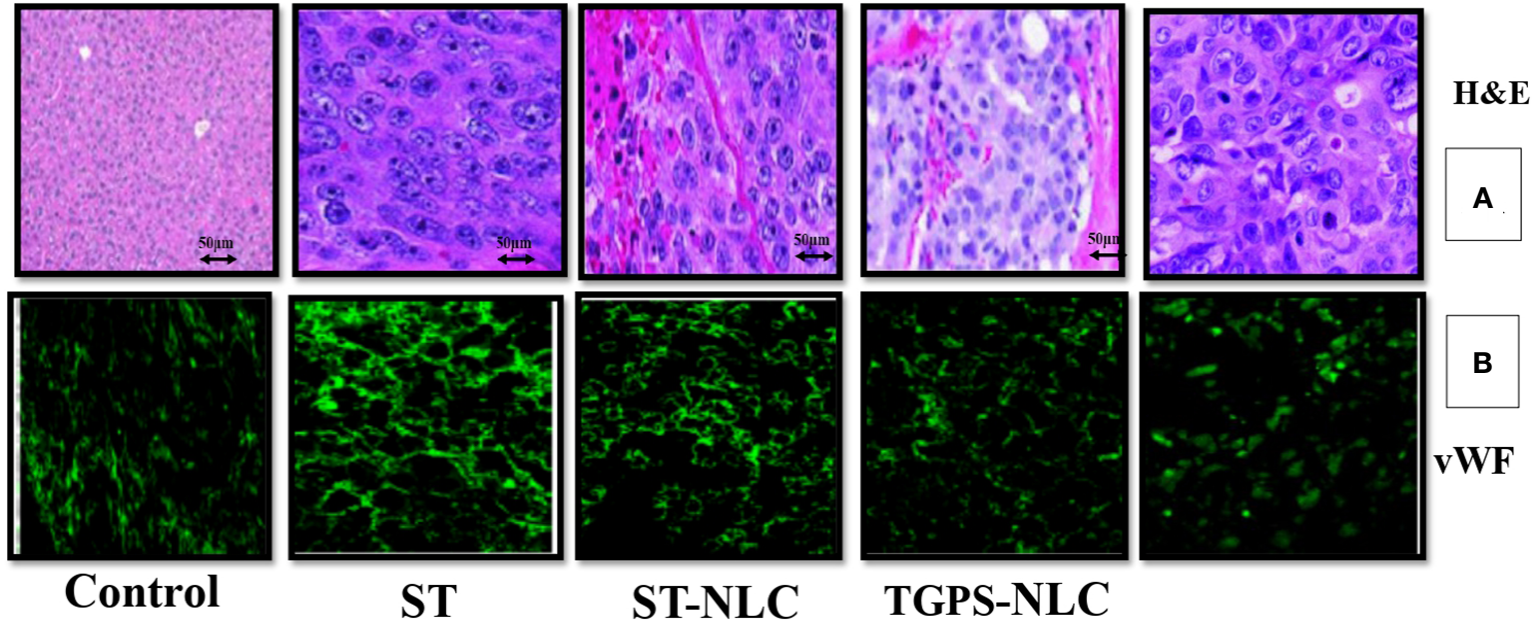
Expression profiles of neovascularization (vWF) in mice tumors **(A)**, Representative pictures of H&E-stained sections from CT-26 xenograft tumors in mice. The expression of neovascularization (vWF expression) was visualized by immunohistochemical study using their specific antibodies. vWF were stained with FITC-conjugated IgG (green) **(B)**. All figures are the representation of three mice tumor sections from each experimental group.

The histopathological analysis by H&E staining of the tumors is summarized in **Table 5** **, **and these data clearly indicated that sorafenib nanoparticles suppressed Xenograft CT-26 induced tumor growth in mice. The immunohistochemical studies showed enhanced expressions of vWF **(** **Figure 9** **)** (an endothelial cell-specific marker). The histopathological analysis by H&E staining of the tumors is summarized in **Table 4**, and these data indicated that sorafenib SLN suppressed xenograft-induced colon cancer growth and angiogenesis in mice.

**Table 5:** Characteristics of xenograft tumors generated in mice.

Tumors Characteristics	Control	Xenograft Group	ST-NLC	ST-TPGS-NLC
Tumor infiltration	++	+++	+	- -
Angiogenesis	- -	+++	+	- -
Tumor cells (Giant)	Less	Plenty	Scanty	Very less
Mitotic condition/Hpf	4-7	12-18	2-5	1-4
Nuclear Polymorphism	Moderate variation	Marked size variation in nucleai	Less nuclear size variation	Small regular uniform

Representation: +: Moderate to poor; ++: Moderate; +++: High; - -: low.

### 3.11 Effects Sorafenib tosylate NPs on cell migration

**Figure 10:**
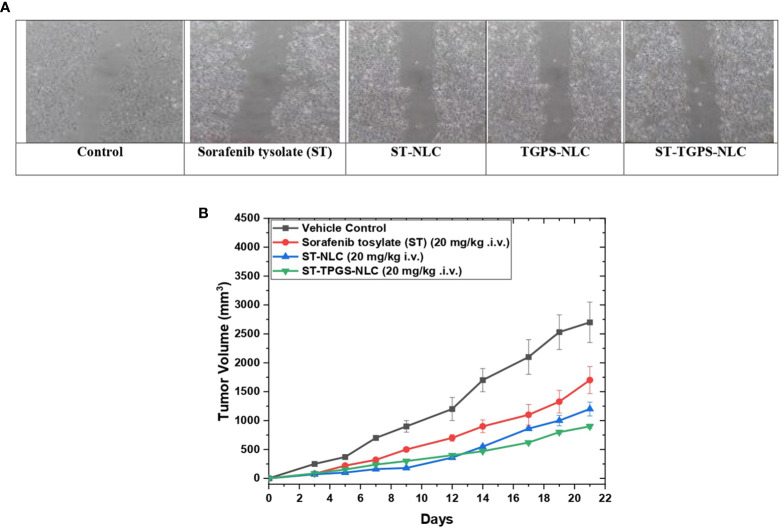
Sorafenib tysolate NPs inhibits cell migration. Effect of Sorafenib tysolate(ST), ST-NLC, TGPS-NLC and ST-TGPS-NLC combinations on cell migration in SW46 PTEN Cell lines **(A)**. Cells were treated for 48h and scratch images were captured at 0, and 24h and analyzed as described in materials and methods. The percent cell migration was expressed as mean ± SD (n=3). where P < 0.05. **(B)**. Tumor regression study in SW48 Cells PTEN cell lines Xenograft model showing changes in relative tumour volume with time (days) of different groups of SCID (severe combined immunodeficiency disorder) mice (n=6) treated with Sorafenib tysolate (ST), ST-NLC and TGPS-NLC (ST equivalent concentration 20 mg/kg, i.v.).

In the SW48 Cells PTEN cell lines, the effects of ST, ST-NLC, TGPS-NLC, and ST-TGPS-NLC on cell migration were investigated. An *in vitro* scratch test was carried out **(** **Figure 10A** **)**. To reduce cytotoxicity, decreased doses of ST, ST-NLC, TGPS-NLC, and ST-TGPS-NLC were chosen for the analysis. Cell migration in the scratch was 100% in the control group after 48 hours, with a complete closing of the gap, while drug therapy alone in cell lines resulted in substantial inhibition of cell migration (P<0.05).

### 3.12 *In vivo* anti-tumor efficacy study

For the anticancer study, sorafenib tosylate (ST), ST-NLC, and ST-TPGS-NLC were injected into the lateral tail veins of mice with SCID (severe combined immunodeficiency disorder). Sorafenib tosylate (ST) (20 mg/kg. i.v.), which is a standard-of-care chemotherapy agent for colorectal cancer (CRC), decreased the size of tumours in animals with established SW48 Cells PTEN tumours by a large amount compared to the normal vehicle control. At 21 days, treatment with sorafenib tosylate (ST) shrinked tumours up to 45.5% compared to the vehicle control group **(** **Figure 10B** **)**. Compared to the vehicle control, the dose of ST-NLC showed that the tumour was 73.22% smaller. but when lyophilized ST-TPGS-NLC (20 mg/kg i.v.) was given to SCID mice, the size of tumours dropped by 91.23% compared to the normal vehicle control group. So, we can say that ST-TPGS-NLC works better on tumours in biological systems. Surprisingly, when ST-TPGS-NLC was injected into animals, they lost weight and had significant anti-tumor effects for a long time. This is an important sign that the drug is rushing after the cancer cells. So, lipid carriers (NLC) systems of sorafenib tosylate (ST) made with D-α-Tocopherol polyethylene glycol 1000 succinate have the benefit of reducing the higher dose-dependent toxicity of anticancer drugs while making the drugs work better against cancer.

## 4 Conclusion

Physicochemical characterization of the nanoscale lipid-based formulations ST-SLN, ST-NLC, and ST-LNE was successfully carried out after they were efficaciously prepared. It was discovered that the size of all plain formulations was less than that of ST-loaded formulations. Nevertheless, a smaller particle size was shaped by the ST-SLN when a higher lipid concentration was used during the preparation process. On the other hand, increasing the concentration of lipids used in the production of ST-NLC and ST-LNE led to an increase in the size of the particles. ST-NLC demonstrated a sustained drug release and contained a significant quantity of lipid. In addition, the optimised ST-NLC formulation was coated with TPGS, which resulted in a slight increase in the particle size while simultaneously demonstrating a slight reduction in drug release. Cytotoxicity testing revealed that the IC50 concentration of pure ST at 48 hours was 14.82 µg/mL, whereas the concentration of ST-TPGS-NLC was only 8.56 µg/mL. In addition, the cytotoxicity tests showed that the TPGS engineered NLC caused a lower number of living cells compared to the pure ST. The findings demonstrated that the use of nanoscale formulations that are lipid-based is an approach that holds great potential for enhancing the therapeutic impact of ST in the treatment of colorectal cancer.

## Data availability statement

The raw data supporting the conclusions of this article will be made available by the authors, without undue reservation.

## Author contributions

SB: conceptualization, supervision, validation, visualization, writing - original draft, communication to the journal. SS writing - review and editing. BP: validation, final approval, supervision. All authors contributed to the article and approved the submitted version.

## Acknowledgments

This initiative would not have been possible without the help and support of Dr. R.S. Gaud, Pharma Section Director, SVKM’s NMIMS Deemed-to-be University, Mumbai, India. Correspondingly, the authors would like to thank DIYA LAB, Mumbai, India, for its logistical and analytical assistance throughout the development of this project.

## Conflict of interest

The authors declare that the research was conducted in the absence of any commercial or financial relationships that could be construed as a potential conflict of interest.

## Publisher’s note

All claims expressed in this article are solely those of the authors and do not necessarily represent those of their affiliated organizations, or those of the publisher, the editors and the reviewers. Any product that may be evaluated in this article, or claim that may be made by its manufacturer, is not guaranteed or endorsed by the publisher.
